# Aortic root evaluation prior to transcatheter aortic valve implantation—Correlation of manual and semi-automatic measurements

**DOI:** 10.1371/journal.pone.0199732

**Published:** 2018-06-28

**Authors:** Barbora Horehledova, Casper Mihl, Chris Schwemmer, Babs M. F. Hendriks, Nienke G. Eijsvoogel, Bastiaan L. J. H. Kietselaer, Joachim E. Wildberger, Marco Das

**Affiliations:** 1 Department of Radiology and Nuclear Medicine, Maastricht University Medical Center, Maastricht, The Netherlands; 2 CARIM School for Cardiovascular Diseases, Maastricht University Medical Center, Maastricht, The Netherlands; 3 Computed Tomography Research & Development, Siemens Healthcare GmbH, Forchheim, Germany; 4 Department of Cardiology, Maastricht University Medical Center, Maastricht, The Netherlands; Universita degli Studi di Bologna, ITALY

## Abstract

**Background:**

Pre-procedural TAVI planning requires highly sophisticated and time-consuming manual measurements performed by experienced readers. Semi-automatic software may assist with partial automation of assessment of multiple parameters. The aim of this study was to evaluate differences between manual and semi-automatic measurements in terms of agreement and time.

**Methods:**

One hundred and twenty TAVI candidates referred for the retrospectively ECG-gated CTA (2^nd^ and 3^rd^ generation dual source CT) were evaluated. Fully manual and semi-automatic measurements of fourteen aortic root parameters were assessed in the 20% phase of the R-R interval. Reading time was compared using paired samples t-test. Inter-software agreement was calculated using the Intraclass correlation coefficient (ICC) in a 2-way mixed effects model. Differences between manual and semi-automatic measurements were evaluated using Bland-Altman analysis.

**Results:**

The time needed for evaluation using semi-automatic assessment (3 min 24 s ± 1 min 7 s) was significantly lower (p<0.001) compared to a fully manual approach (6 min 31 sec ± 1 min 1 sec). Excellent inter-software agreement was found (ICC = 0.93 ± 0.0; range:0.90–0.95).

The same prosthesis size from manual and semi-automatic measurements was selected in 92% of cases, when sizing was based on annular area. Prosthesis sizing based on annular short diameter and perimeter agreed in 99% and 96% cases, respectively.

**Conclusion:**

Use of semi-automatic software in pre-TAVI evaluation results in comparable results in respect of measurements and selected valve prosthesis size, while necessary reading time is significantly lower.

## Introduction

Transcatheter aortic valve implantation (TAVI) provides a minimally invasive therapeutical option to patients with severe aortic valve stenosis who are not eligible for conventional valve replacement [1–8]. Precise assessment of aortic root prior to the intervention is fundamental for patient’s outcome. It allows for recognition of suitable patients, selection of the correct valve for replacement, and therefore minimizes the risk of peri-procedural complications [9].

Need for comprehensive imaging with high spatial and temporal resolution, which is not impaired by dynamic movement of aortic root during cardiac cycle is the reason why multidetector row CT (MDCT) has been addressed as the method of choice in pre-TAVI evaluation [10–13]. MDCT assessment of aortic root was recently also considered a predictive factor for severity of paravalvular leakage, which further supports the role of MDCT in TAVI planning [11, 14].

However, tedious manual assessment of aortic root dimensions is highly sophisticated and therefore requires an experienced reader who is able to reliably extract necessary data from the scan. Semi-automatic software is designed to determine anatomical structures of aortic annulus on MDCT scan, allowing partial automation of measurements and in this respect simplifying the assessment, hereby making TAVI planning faster.

Similar assisting software tools, with different levels of automation, have been previously described in the limited extent of four or five aortic root dimensions [15, 16], however, in our clinical practice fourteen aortic root parameters are routinely assessed in each TAVI candidate, following the expert consensus guidelines on MDCT imaging before TAVI [17, 18].

The aim of this study was to evaluate the agreement and time effectiveness of a prototype of a semi-automatic software with the standard manual assessment in aortic root evaluation prior to TAVI.

## Materials and methods

### Study population and data collection

Between April 2014 and April 2016, 120 patients with severe AS were retrospectively evaluated. All patients were referred from the cardiology outpatient department for pre-interventional assessment of aortic root dimensions and peripheral arteries. Patients with a history of valve replacement were excluded from the data analysis.

All procedures performed in studies involving human participants were in accordance with the ethical standards of the institutional and/or national research committee and with the 1964 Helsinki declaration and its later amendments or comparable ethical standards. The approval for this study was obtained from the Institutional Review Board and the local medical ethical research committee (METC). Due to the retrospective nature of this study a waiver of written informed consent was issued by the Institutional Review Board. The data were coded and analyzed anonymously. The local METC (METC—Maastricht University Medical Center) reference number is 15-4-202.

### Imaging protocol & post-processing

Retrospectively ECG-gated spiral MDCT of the aortic root was performed on a 2^nd^ and 3^rd^ generation Dual source CT scanner (Somatom Definition Flash & Somatom Force, Siemens Healthcare GmbH, Forchheim, Germany) following an established institutional protocol [19]. Images were reconstructed at every 10% of the R–R interval with individually adapted field of view (FOV) at 0.6 mm slice thickness with an increment of 0.4mm using iterative reconstruction (strength 3) and l26f kernel. Measurements were performed with dedicated post-processing software (Syngo.via^™^ Siemens Healthcare GmbH, Forchheim, Germany).

### Objective CT image quality

CT image quality was quantified, in terms of attenuation in Hounsfield Units (HU), standard deviation (SD), signal-to-noise ratio (SNR) and contrast-to-noise ratio (CNR). Vascular attenuation was assessed with manually placed circular regions of interest (ROI) at the level of sinotubular junction. For noise and signal estimation another ROI was placed in the perivascular tissue in the left ventricular myocardium. CNR was calculated as vascular attenuation minus perivascular tissue attenuation, divided by the SD of the perivascular tissue attenuation. SNR was calculated as vascular attenuation divided by the SD of vascular attenuation [19]. Image quality was considered diagnostic with vascular attenuation values > 200 HU [20, 21] and CNR > 3 [22].

### Subjective CT image quality

The subjective image quality, in terms of presence or absence of cardiac motion artifacts, was qualitatively evaluated by an experienced cardiovascular radiologist (CM) using previously published 4-point Likert scale [23]: grade 1 –non-diagnostic: impaired image quality that precluded appropriate evaluation of the aortic root due to severe motion artifacts; grade 2—diagnostic: reduced image quality due to motion artifacts, but sufficient to assess aortic root dimensions; grade 3—good: presence of motion artifacts, but fully preserved ability to reliably assess aortic annulus dimensions; grade 4—excellent: complete absence of motion artifacts. Cardiac motion artifacts were defined as beam-hardening, stair-stepping, blurring, ghosting, streaking, linear bands, areas of isolated or multiple discontinuity or dark shadows [24].

### Aortic annulus and leaflet calcifications

The presence of aortic annulus calcification was qualitatively analyzed and graded in consensus by 2 observers (BH, CM) using a 5-point Likert scale: grade 0: absent annulus calcification; grade 1—minimal (< 25% of total annulus circumference); grade 2—mild (25–50% of total annulus circumference); grade 3—moderate (50–75% of total annulus circumference); grade 4—severe (75–100% of total annulus circumference) [25]. The aortic valve leaflet calcifications were graded as present or absent.

### Time tracking

**Fig 1:**
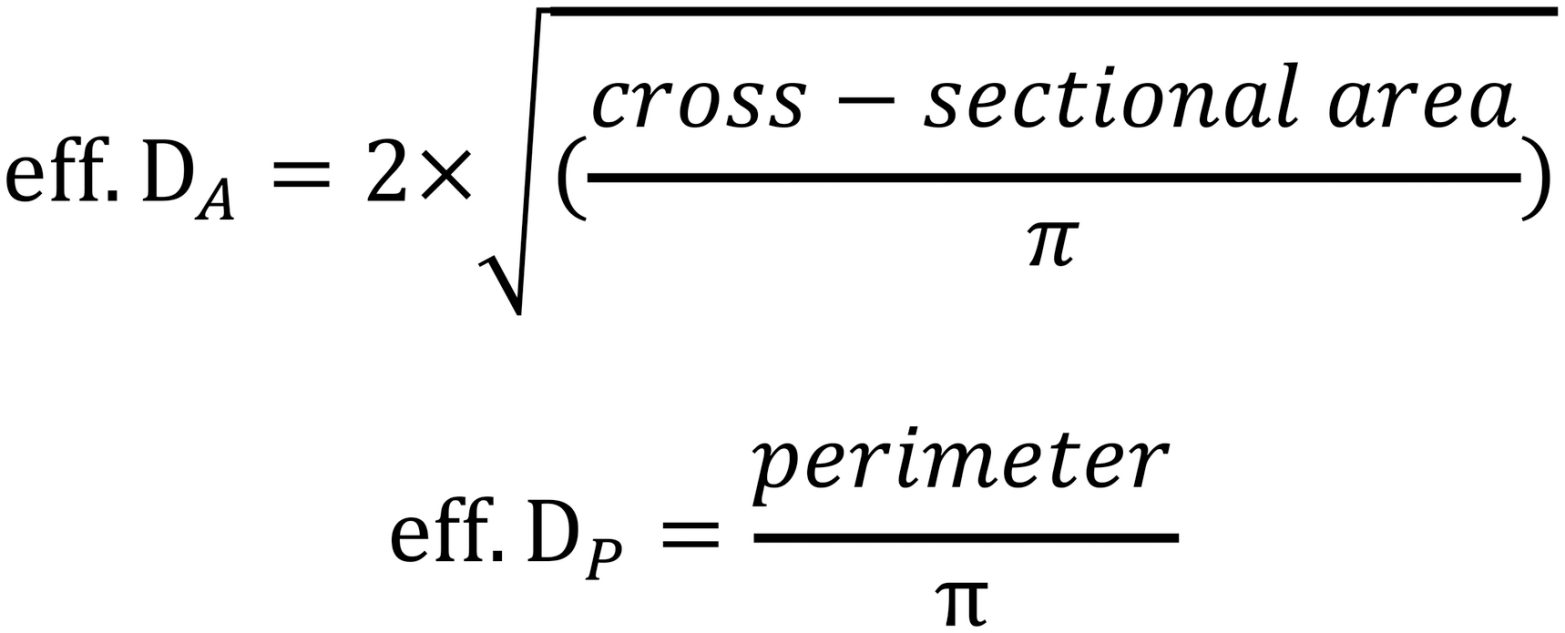
Formulas used for calculation of effective diameter. eff. D_A_ = effective diameter derived from aortic annulus area, eff. D_P_ = effective diameter derived from aortic annulus perimeter.

Case evaluation time was recorded within each assessment. A time tracking macro tool (programmed in Microsoft Excel, Microsoft corporation, Redmond, WA, USA) was built in the electronic worksheet. Therefore pre-TAVI measurements and reading time were recorded simultaneously to assure uniformity and accuracy of time measurements, regardless of method or reader performing the evaluation. The time tracking tool assessed the software loading time, describing the time before measurements could be carried out and time used for assessment of aortic root measurements as individual time points within evaluation. Mean necessary reading time was established for manual and semi-automatic approach. In manual assessment, the effective diameters derived from annular area and perimeter (eff. D_A_ and eff. D_P_, respecitvely) had to be additionally calculated with dedicated formulas (Fig 1), as these values are not directly available during manual analysis. However, the time needed to calculate effective diameters is not significant and was therefore disregarded.

Software loading time was tracked, but excluded from further evaluation because semi-automatic measurements were performed in preclinical interface.

### Measurements

Pre-TAVI measurements were performed in fully manual and semi-automatic approach in each patient in the 20% phase of cardiac cycle, according to previous literature [26]. Results of earlier evaluation (manual or semi-automatic; inter-observer) were blinded during the subsequent data analysis with the remaining method. The following dimensions of the aortic root were assessed, in accordance to the expert consensus guidelines of the Society of Cardiovascular Computed Tomography (SCCT) [17]:

- short and long annulus diameter
- cross-sectional area of aortic annulus (defined as an area within an oval or ring formed by linking the most basal portions of the leaflet attachments)
- aortic annulus perimeter
- distance from the aortic annulus to the center of the ostium of the left and right coronary artery
- widest diameter at the sinotubular junction
- aortic root diameter at the level of the left and right coronary artery ostium
- widest portion of the coronary sinuses diameter
- length of left and right aortic valve leaflet
- eff. D_P_ and eff. D_A_

Agreement between manual and semi-automatic method and inter-reader agreement in semi-automatic approach were estimated in all measured parameters. In patients suitable for TAVI procedure the TAVI prosthesis size (Edwards Sapien Valve, Edwards Lifesciences Corp, Irvine, USA) was theoretically selected according to published recommendations [17, 27]. The agreement in selected prosthesis size derived from manual and semi-automatic method was evaluated.

### Manual measurements

**Fig 2:**
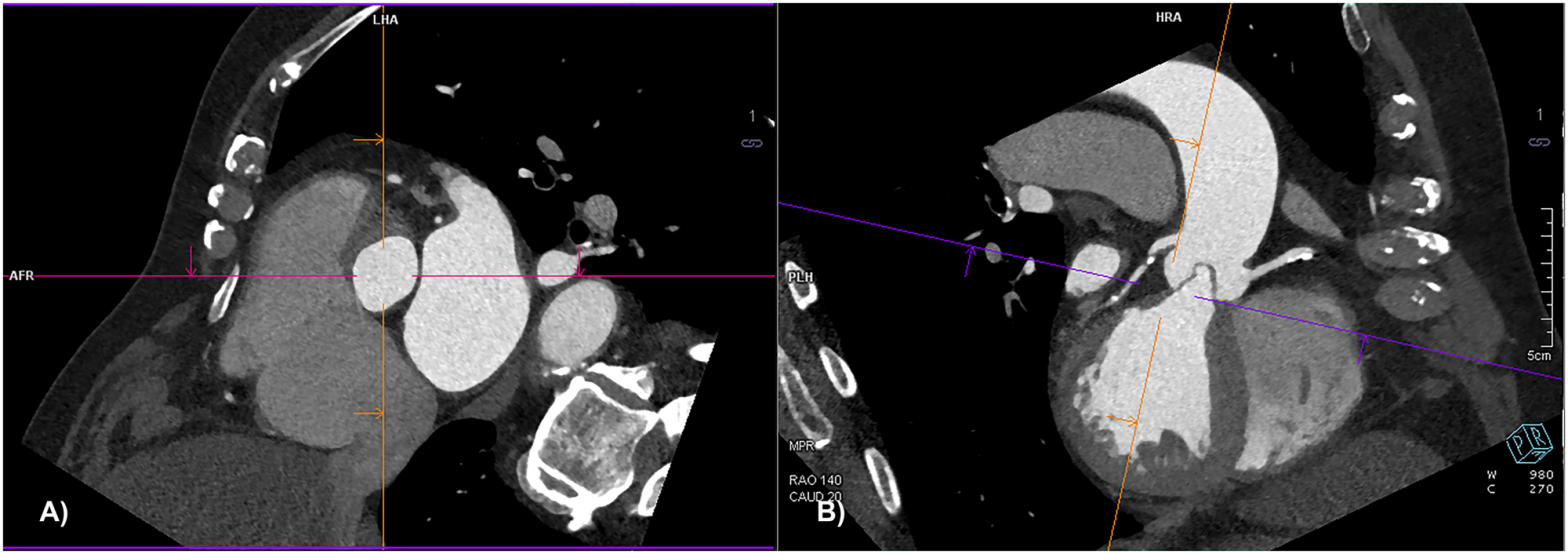
CT TAVI planning, Syngo.via^™^ VB10A Siemens, Siemens Healthcare GmbH, Forchheim Germany. (A) The oblique transversal plane crossing the most basal attachments of the aortic valve leaflets. (B) The oblique sagittal (coronal) plane of the aortic valve showing leaflets.

The manual assessment was performed with a dedicated software workflow (CT TAVI planning, Syngo.via^™^ VB10A Siemens). The 20% phase of the R-R interval was selected. Image data were manipulated in order to create an oblique transversal plane corresponding to the aortic annulus, crossing the most basal attachments of the aortic leaflets (Fig 2A), where aortic annulus diameters, area and perimeter were assessed. Eff. D_A_ and eff. D_P_ were calculated manually. An oblique transversal plane perpendicular to the course of the aorta was used to measure the dimensions of the widest portion of aortic root, the diameter at the level of sinotubular junction and at the level of coronary artery ostia. The oblique sagittal or coronal plane was used for the measurement of the distances from aortic annulus to the coronary artery ostia, and the length of the aortic leaflets (Fig 2B). Manual assessment was performed by a research fellow specially trained for this analysis (BH), with one year of experience in TAVI planning.

### Semi-automatic measurements

**Fig 3:**
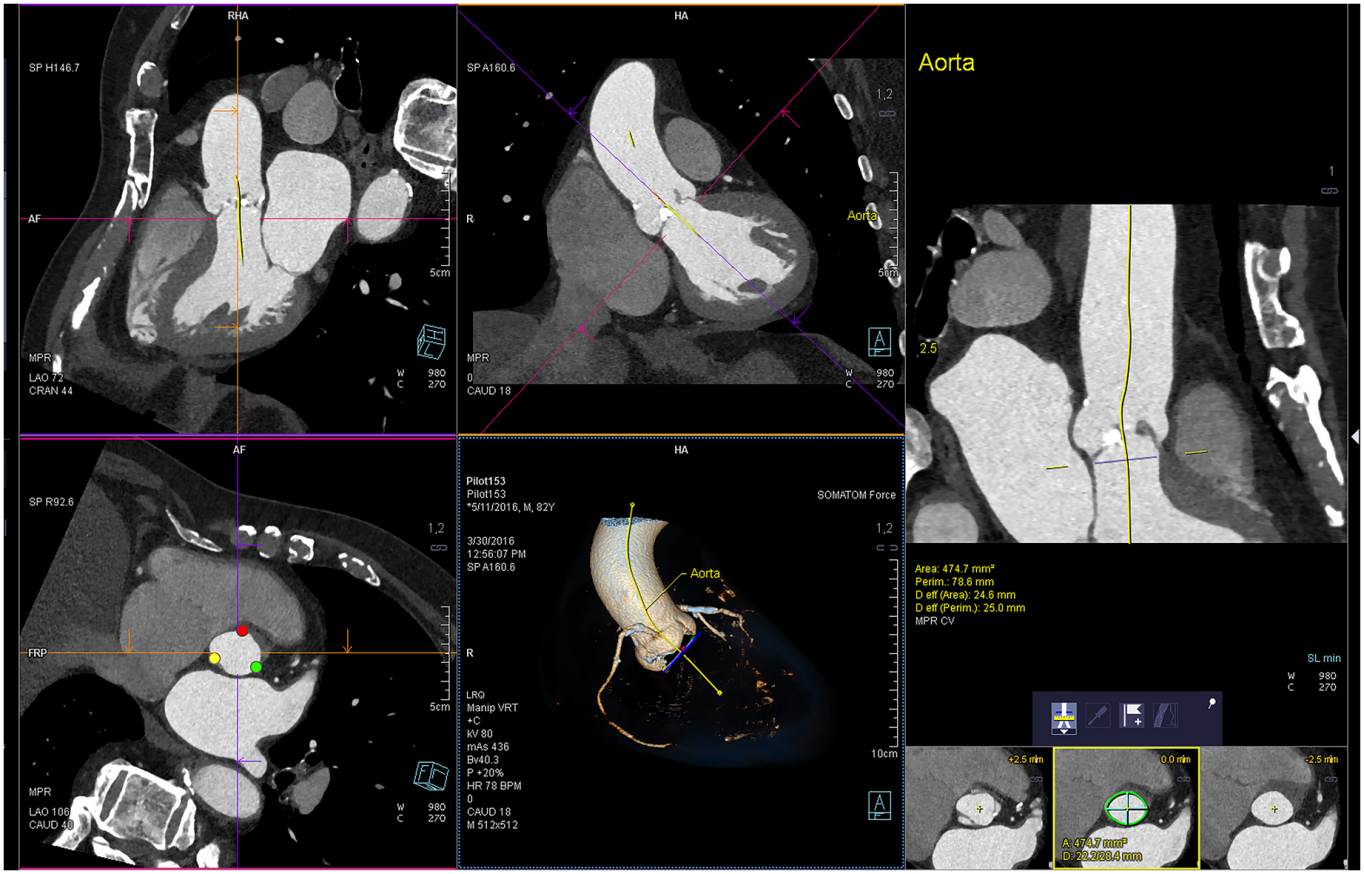
Automatic aortic annulus plane of prototype version of syngo.CT Cardiac Planning. Syngo.via^™^ VB20A, Siemens Healthcare GmbH, Forchheim Germany.

Semi-automatic measurements were performed with a prototype version of syngo.CT Cardiac Planning software (Syngo.via^™^ VB20A, Siemens). The semi-automatic software automatically detects the three most basal attachment points of aortic valve leaflets, therefore opens the user interface directly in the annulus plane in the endsystolic phase. Measurements of short and long annulus diameter, annular area and perimeter are fully automatic. Likewise, eff. D_A_ and eff. D_P_ are calculated by the software and presented instantly. A centerline is computed through the aortic root and ascending aorta perpendicularly to the estimated aortic annulus plane. A dedicated tool (diameter ruler) displays automatically minimum and maximum diameters, perimeter, cross-sectional area and effective diameters at respective levels of the aortic root and ascending aorta while scrolling along the centerline. This allows for semi-automatic assessment of aortic root diameters (widest portion of aortic sinuses, diameter at sinotubular junction, diameter at the level of coronary ostia). Another dedicated tool assists only with image manipulation in order to visualize both left and right coronary ostia in one oblique sagittal plane (see Fig 3). Measurements of the distance from the aortic annulus to the coronary ostia as well as the length of coronary leaflets are then assessed manually.

In addition, the reproducibility of semi-automatic measurements was evaluated. A research fellow specially trained in pre-TAVI evaluation with one year of experience (BH; reader 1) and a cardiovascular radiologist (CM; reader 2) performed the semi-automatic measurements independently and fully blinded to each other, in all 120 cases.

### Statistical analysis

Dimensions of each anatomical structure and time needed for evaluations were evaluated using descriptive statistics (mean ± SD) calculated with independent samples t-test. Correlation and agreement between the manual and semi-automatic method were determined with the use of the paired samples t-test and Bland-Altman methods with 95% confidence interval (CI; mean difference ± 1.96 x SD). Inter-observer agreement was calculated using the Intraclass correlation coefficient (ICC) in a 2-way mixed effects model Bland-Altman methods with 95% CI. Statistical analysis was conducted using Statistical Package for Social Sciences version 23.0 (SPSS Inc., Chicago, IL, USA). All p-values are 2-sided, and a p-value < 0.05 was considered statistically significant.

## Results

### Baseline characteristics

Baseline characteristics are listed in Table 1. The study population consisted of 68 male and 52 female patients with an average age of 78 ± 8 years.

**Table 1:** Baseline characteristics.

Baseline characteristics	Mean	Range
Age [years]	78 ± 8	43–90
Gender	Male / Female	68 (57%) / 52 (43%)
Height [cm]	167 ± 10	135–193
Weight [kg]	77 ± 19	45–150
BMI [kg/m^2^]	28 ± 6	16–46

cm = centimeter; kg = kilogram; m = meter

### Objective CT image quality

All examinations were of diagnostic quality, allowing aortic valve evaluation in all 120 included cases. The mean attenuation value at sinotubular junction was 426 ± 108 HU, with mean SNR 12 ± 6 and mean CRN 11 ± 5.

### Subjective CT image quality

Cardiac motion artifacts were completely absent (grade 4) in 101 images (84%), non-significant motion artifacts (grade 3) occurred in 19 scans (16%). No scans presented with non-diagnostic or diagnostic level of artifacts (grade 1 or 2).

### Aortic annulus and leaflet calcifications

The annulus calcification was present in total of 118 cases (98%). In 105 patients (88%) was the extent of annular calcification graded as minimal and in 13 as mild (18%). In 5 cases (4%) the aortic annulus calcification extended caudally into the left ventricular outflow tract. The aortic valve leaflets were calcified in all assessed patients.

### Time tracking

All time tracking values in all established time points are stated in Table 2. Mean necessary reading time for manual measurements was 6 min 31 s ± 1 min 1 s. Mean necessary reading time for semi-automatic measurements were 3 min 24 s ± 1 min 7 s.

**Table 2:** Time tracking of manual and semi-automatic measurements.

**Manual assessment:**	**Mean ± SD**	**p—value**
• Total Reading time	7 min 36 s ± 1 min 7 s	<0.001
• Loading time	1 min 5 s ± 0 min 23 s	<0.001
• Measurements time	6 min 31 s ± 1 min 1 s	<0.001
**Semi-automatic assessment:**		
• Total Reading time	3 min 55 s ± 1min 19 s	<0.001
• Loading time	0 min 31 s ± 0 min 21 s	<0.001
• Measurements time	3 min 24 s ± 1 min 7 s	<0.001

Total reading time = loading time + measurements time; Loading time = time before measurements could be carried; Measurement time = time for assessing evaluated measurements; SD = standard deviation

### Manual and semi-automatic measurements

**Fig 4:**
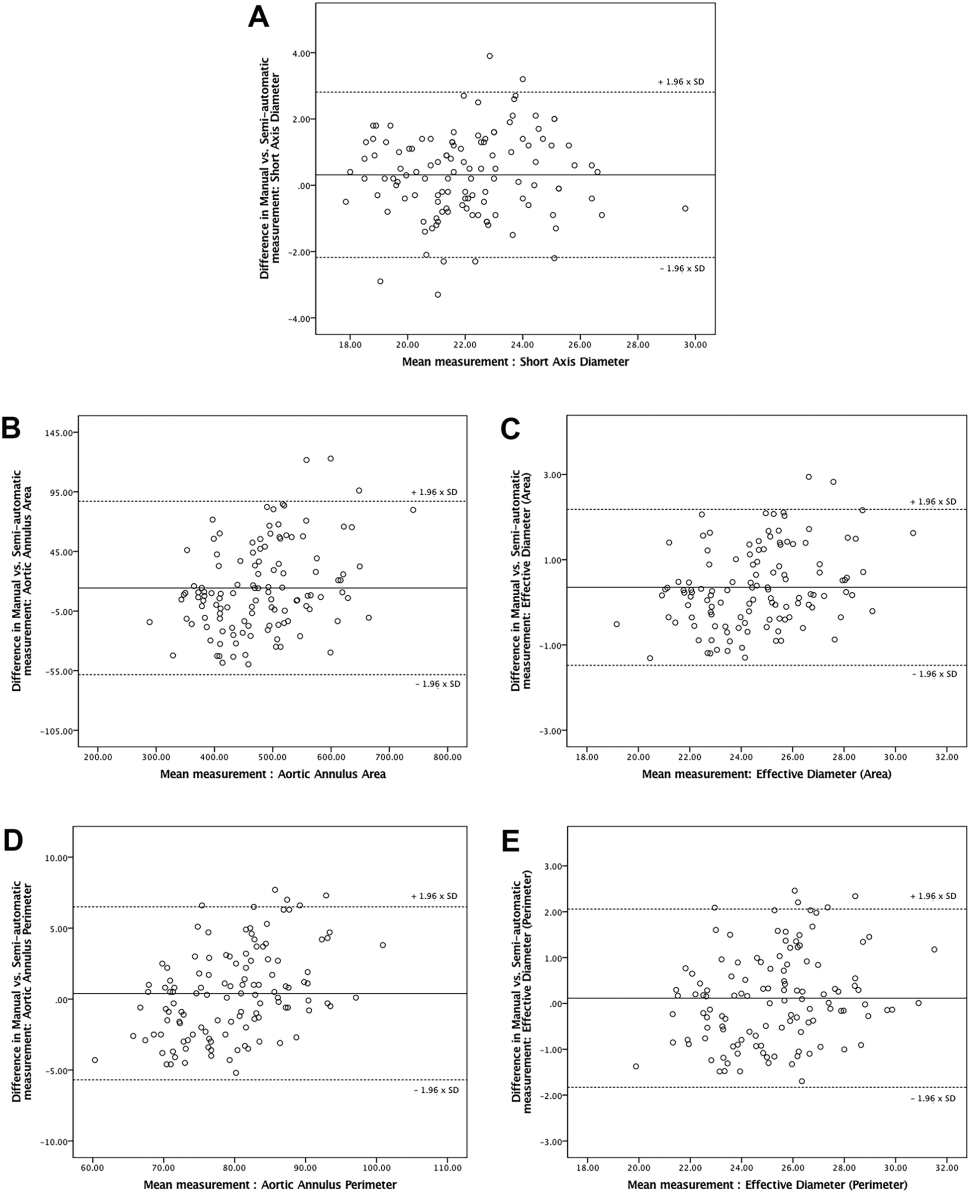
Bland-Altman plots demonstrating agreement between fully manual and semi-automatic MDCT measurements of annular dimensions commonly used in prosthesis size selection shown with 95% confidence interval. The middle line presents the mean difference (expressed in mm for aortic annulus diameters and perimeter, mm^2^ for aortic annulus area) and the upper and lower lines represent 95% confidence interval. (A) Short Axis Diameter; (B) Aortic Annulus Area; (C) Effective Diameter (Area); (D) Aortic Annulus Perimeter; (E) Effective Diameter (Perimeter).

All manual and semi-automatic measurements are stated in the S1 Table. Excellent inter-software agreement was found between manual and semi-automatic method (ICC = 0.93 ± 0.02 for parameters with possible semi-automatic assessment and ICC = 0.83 ± 0.17 for all accessed dimensions; Table 3) [28]. The lowest ICC between manual and semi-automatic assessment was established for distance from aortic annulus to left and right coronary ostium (ICC = 0.46 and 0.51, respectively). The highest agreement was found in measurement of area, perimeter and effective diameters (ICC = 0.95). The mean difference between manual and semi-automatic assessment of short diameter, annular area and perimeter, eff. D_A_ and eff. D_P_ were 0.31 mm (95% CI: -2.18 to 2.81), 14.38 mm^2^ (95% CI: -58.37 to 87.13), 0.39 mm (95% CI: -5.70 to 6.49), 0.35 mm (95%: -1.48 to 2.18), and 0.12 mm (95% CI: -1.83 to 2.06), respectively (Fig 4A–4D).

**Table 3:** Intraclass correlation agreement (ICC) between manual and semi-automatic approach and inter-observer agreement in semi-automatic approach.

	ICC manual & semi-automatic measurements	ICC semi-automatic measurements	
**Short annulus diameter**	0.911	0.967	*Automated measurements*
**Long annulus diameter**	0.916	0.965	
**Annulus area**	0.945	0.98	
**Eff. D**_**A**_	0.947	0.984	
**Perimeter**	0.950	0.984	
**Eff. D**_**P**_	0.950	0.983	
**Widest portion of aortic root**	0.92	0.988	*Semi-automatic measurements*
**Diameter—Left ostium**	0.902	0.977	
**Diameter—Right ostium**	0.898	0.963	
**Diameter—sinotubular junction**	0.918	0.970	
**Dist. annulus to the left ostium**	0.464	0.918	*Manual measurements*
**Dist. annulus to the right ostium**	0.513	0.883	
**Left leaflet length**	0.696	0.652	
**Right leaflet length**	0.734	0.649	

dist. = distance; eff. D_A_ = diameter derived from aortic annulus area; eff. D_P_ = diameter derived from aortic annulus perimeter; eff. = effective; ICC = Intraclass correlation coefficient

**Fig 5:**
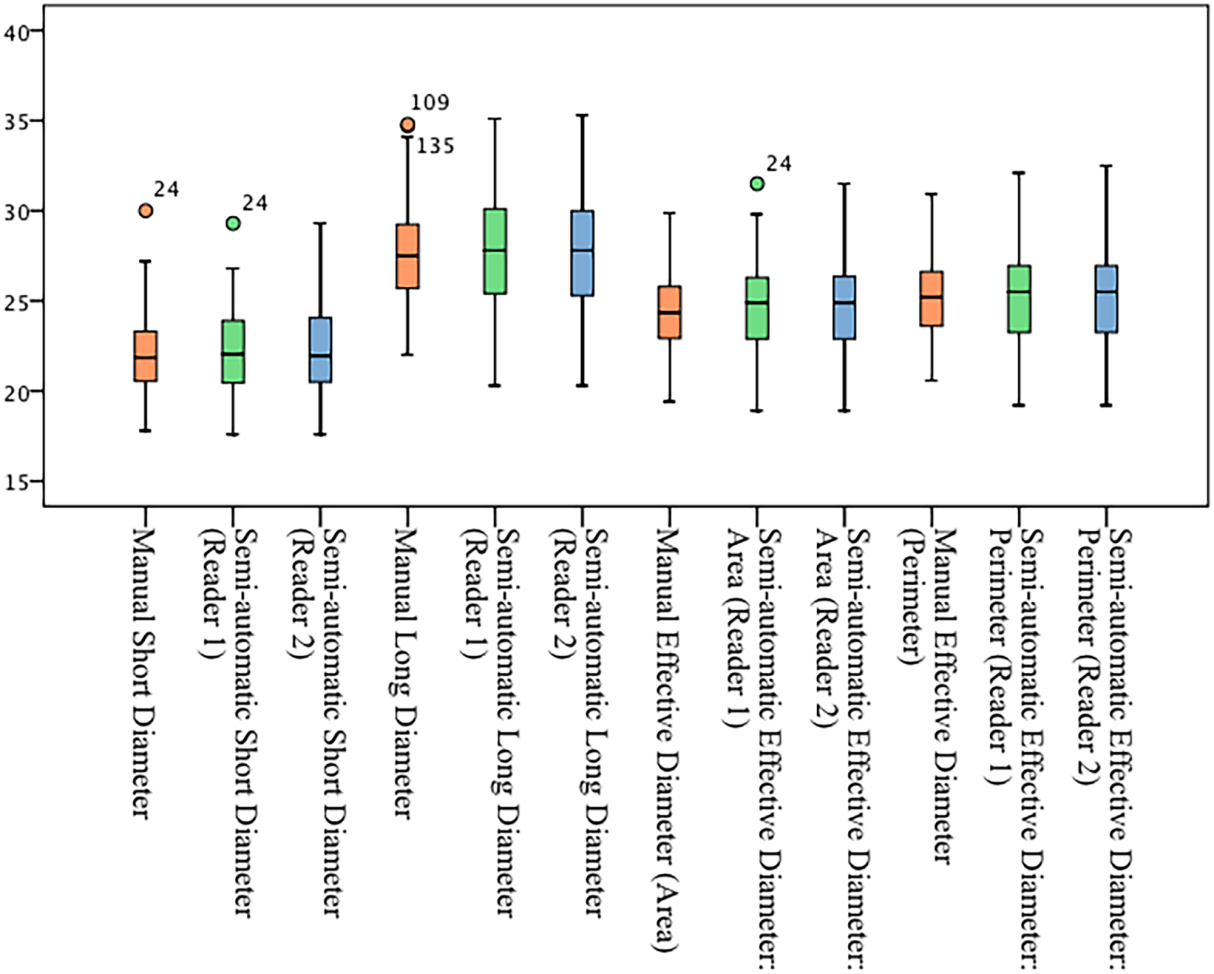
Aortic annulus diameter measured manually and with semi-automatic software. mm = millimeter.

Excellent inter-observer correlation in semi-automatic software was found in all assessed dimensions (ICC = 0.92 ± 0.12). The lowest observed mean ICC was found for the length of the cusps (ICC = 0.65) and the highest was found for the widest portion of the coronary sinuses diameter (ICC = 0.99). The mean inter-observer difference was ≤ -0.1 mm for annular diameters, -0.2 mm (95% CI: -4.02 to 3.62,) for annular perimeter and—3.3 mm^2^ (95% CI: -54.59 to 47.99) for annular area (Fig 5).

### TAVI prosthesis size selection

**Fig 6:**
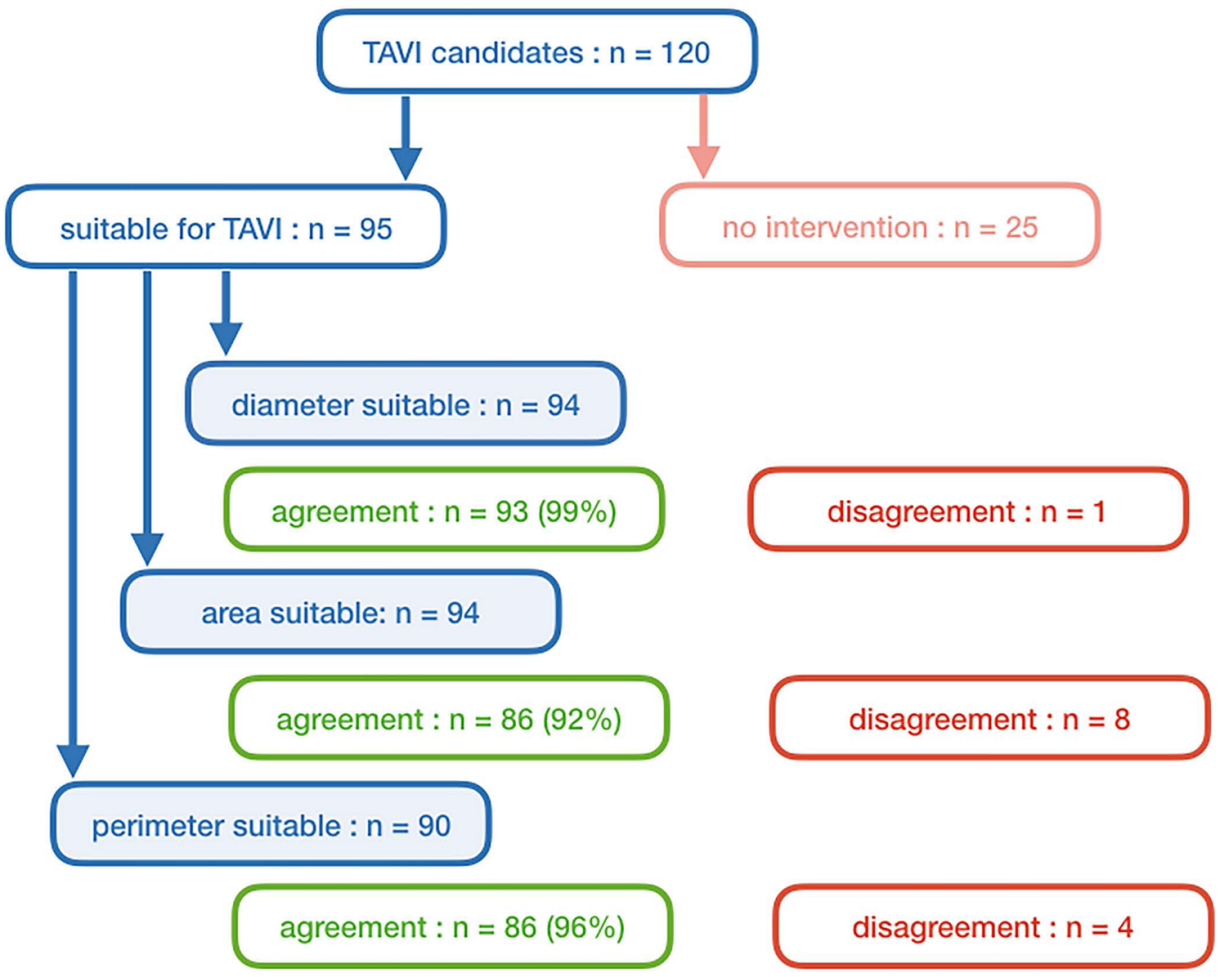
Theoretical TAVI prosthesis size selection (Edwards SapienValve). n = number; TAVI = transcatheter aortic valve implantation. Theoretical prosthesis size selection according to recommendations for Edwards SapienValve (Edwards Lifesciences Corp, Irvine, USA).

Ninety-five patients finally were eligible for TAVI procedure (Fig 6). Theoretical prosthesis sizing based on short annular diameter was possible in 94 cases, as appropriate prosthesis size is not available for one patient with annulus diameter of 29.1 mm. Prosthesis sizing derived from short annular diameter resulted in size agreement in 93 cases (99%). In one case the manual assessment suggested TAVI prosthesis one size larger compared to the semi-automatic assessment. The same prosthesis size was suggested in 86 cases (92%) when sizing was based on annular area and in 86 cases (96%) based on annular perimeter.

## Discussion

This study demonstrates that the evaluated semi-automatic software provides reliable aortic root measurements with an excellent inter-observer agreement. The prototype of the semi-automatic software is able to automatically recognize aortic annulus dimensions with an excellent agreement to manual assessment (ICC = 0.91–0.95), while the semi-automatic software is significantly less time consuming.

Precise measurements of aortic annulus dimensions are not only essential for selection of patients suitable for TAVI procedure, they are also crucial for particular selection of prosthesis size, which directly impacts patient’s outcome [9, 14]. Special attention was therefore paid to the annular dimensions suggested to be used in prosthesis size selection according to the industry guidelines. The mean difference between manual and semi-automatic measurements was ≤ 0.35 mm for annular diameters, 0.39 mm for annular perimeter and 14.38 mm^2^ for annular area with an excellent ICC of 0.91 or higher. Even though the difference was statistically significant for short aortic annulus, annular area and eff. D_A_, it should not be considered as clinically relevant according to previous literature [29]. This assumption is further supported with a 92–99% agreement between manual and semi-automatic assessment, when hypothetical valve sizing was applied in this study. Particularly, when agreement in suggested valve size of 59.4% was reported for manual measurements performed by different readers in study by Lou et al. in 2015 [15].

The moderate agreement in the annulus to coronary artery ostium distance (ICC = 0.46; 0.51) can be explained by the different data segmentation between methods. In a manual approach, measurements are carried out in a coronal plane, whereas the semi-automatic software arranges the aortic root along a calculated centerline, perpendicular to the aortic annulus plane, slightly affecting ostial position. However, comparable moderate agreement (ICC 0.48; inter-rater difference of up to 6.7 mm) was previously also reported for inter-rater agreement in manual assessment [30]. More importantly, the mean difference of 3.5 mm in annulus-ostium distance was previously considered acceptable in study by Watanabe et al. [16].

Nevertheless, the calculated centerline in semi-automatic software also offers a higher confidence while assessing the semi-manual measurements of aortic root diameters (ICC = 0.90–0.92). A dedicated tool “diameter ruler” works along the calculated centerline in the curved planar reformation (CPR) segment, directly presenting the minimum and maximum diameter at respective level of aortic root while scrolling. This feature allows the reader to distinguish the true maximum diameter faster and in more sophisticated way in comparison to manual assessment, where multiple manual measurements at different levels of aortic root have to be performed.

Therefore, apart from the increased level of user convenience regarding measurements, semi-automatic software also demonstrated a greater efficiency in terms of time consumption. Evaluating pre-TAVI scans with semi-automatic software was approximately 3 minutes faster, allowing to process twice as many pre-TAVI datasets, while delivering comparable results. Manual assessment was slower mainly due to need for initial data manipulation, in order to recognize aortic annulus plane.

Inter-observer agreement in this study was nearly perfect using the semi-automatic software, which offered reproducible results in terms of measurements, without necessarily reflecting the experience of the reader. Data segmentation, in matter of recognition or position of basal insertions of aortic valve cusps, may not satisfy the reader completely in some cases (e.g. due to poor image quality, etc.) [15], however, only in 4 out of the 120 cases in this study the reader decided to redefine the basal hinge points.

A limitation of this study was that only two readers were able to perform semi-automatic measurements on the prototype version of software due to limited availability of workstations with the prototype software, and only one of these readers performed the manual measurements in all 120 cases. This study aimed to evaluate inter-software agreement and reproducibility rather than accuracy, because of the absence of true reference standard, such as direct anatomic measurement. Correlation of pre-TAVI measurements to post-interventional outcome was not possible in most cases, as our institution serves as a TAVI referral center and many patients undergo follow up in an external facilities.

## Conclusion

The use of semi-automatic software in pre-TAVI evaluation results in comparable outcome in respect of measurements and selected valve prosthesis size in comparison to a manual approach, while the necessary reading time is significantly lower.

Inter-observer agreement of semi-automatic measurements is excellent, proposing a possibility to standardize aortic annulus measurements, with a high confidence level regardless of reader experience.

## Supporting information

S1 Table Pre-TAVI evaluation assessment with manual and semi-automatic approach expressed as mean values ± SD and mean difference with 95% confidence interval from Bland-Altman analysis. CI = confidence interval; man = manual; mm = millimeter; SA = semi-automatic method; SA1 = semi-automatic method performed by readered 1; SA2 = semi-automatic method performed by readered 2; sign. = significance.
(DOCX)

## Abbreviations

BH: reader 1
CI: confidence interval
CM: reader 2
CNR: contrast-to-noise ratio
CPR: curved planar reconstruction
CT: computed tomography
ED: effective diameter
eff D_A_: effective diameter derived from aortic annulus area
eff D_P_: effective diameter derived from aortic annulus perimeter
FOV: field of view
HU: Hounsfield Units
ICC: Intraclass correlation coefficient
LBBB: left bundle branch block
MDCT: multidetector row CT
METC: medical ethical research committee
ROI: region of interest
SCCT: Society of Cardiovascular Computed Tomography
SD: standard deviation
SNR: signal-to-noise ratio
SPSS: Statistical Package for Social Sciences
TAVI: transcatheter aortic valve implantation
