# Supplementary material for: Aortic root evaluation prior to transcatheter aortic valve implantation—Correlation of manual and semi-automatic measurements
Source: PLoS One. 2018 Jun 28;13(6):e0199732. doi: 10.1371/journal.pone.0199732 (PMC6023104; doi:10.1371/journal.pone.0199732)
Supplement: S1 Table — CI = confidence interval; man = manual; mm = millimeter; SA = semi-automatic method; SA1 = semi-automatic method performed by readered 1; SA2 = semi-automatic method performed by readered 2; sign. = significance. (DOCX) [file pone.0199732.s001.docx]

S1 Table. Pre-TAVI evaluation assessment with manual and semi-automatic approach expressed as mean values ± SD and mean difference with 95 % confidence interval from Bland-Altman analysis

|  | **Manual**  **[mm]** | **Semi - automatic (SA1) [mm]** | **Semi - automatic (SA 2) [mm]** | Mean difference  (SA1-man) **[mm]** | 95 % CI | Sign. (2 – tailed) | Mean difference  (SA1-SA2)  **[mm]** | 95 % CI | Sign. (2 – tailed) |
| --- | --- | --- | --- | --- | --- | --- | --- | --- | --- |
| **Short annulus diameter** | 21.9 ± 2.2 | 22.3 ± 2.3 | 22.4 ± 2.4 | 0.31 | -2.18- 2.81 | p = 0.008 | -0.1017 | -1.73 -1.53 | p = 0.184 |
| **Long annulus diameter** | 27.7 ± 2.7 | 27.7 ± 3.0 | 27.7 ± 3.0 | 0.02 | -3.12 -3.15 | p = 0.918 | -0.0292 | -2.20 - 2.14 | p = 0.773 |
| **Annulus area** | 472 ± 80 | 486 ± 92 | 490 ± 96 | 14.38 | -58.37 - 87.13 | p < 0.001 | -3.3000 | -54.59 -47.99 | p = 0.170 |
| **Effective diameter (area)** | 24.4 ± 2.1 | 24.8 ± 2.3 | 24.9 ± 2.4 | 0.35 | -1.48 - 2.18 | p < 0.001 | -0.0733 | -1.24 - 1.10 | p = 0.180 |
| **Perimeter** | 79.1 ± 6.8 | 79.5 ± 7.5 | 79.7 ± 7.8 | 0.39 | -5.70 - 6.49 | p = 0.168 | -0.2000 | -4.02 -3.62 | p = 0.263 |
| **Effective diameter (Perimeter)** | 25.2 ± 2.2 | 25.3 ± 2.4 | 25.4 ± 2.5 | 0.12 | -1.83 - 2.06 | p = 0.201 | -0.0650 | -1.29 - 1.16 | p = 0.255 |
| **Distance to left ostium** | 16.8 ± 2.5 | 13.5 ± 2.8 | 13.8 ± 2.5 | -3.27 | -8.36 - 1.82 | p < 0.001 | -0.3250 | -3.16 - 2.51 | p = 0.015 |
| **Distance to right ostium** | 20.2 ± 3.3 | 16.4 ± 3.2 | 16.3 ± 3.0 | -3.80 | -9.57 – 1,97 | p < 0.001 | 0.1018 | -3.82 -4.02 | p = 0.578 |
| **Widest portion of aortic root** | 36.6 ± 4.3 | 35.1 ± 4.1 | 35.0 ± 4.0 | -1.50 | -5.04 - 2.04 | p < 0.001 | 0.1892 | -1.48 - 1.86 | p = 0.016 |
| **Diameter at left ostium** | 35.7 ± 4.4 | 34.1 ± 4.0 | 34.2 ± 4.1 | -1.62 | -5.67 - 2.43 | p < 0.001 | -0.1000 | -2.49 -2.29 | p = 0.371 |
| **Diameter at right ostium** | 33.0 ± 4.6 | 33.0 ± 4.0 | 32.6 ± 3.8 | 0.03 | -5.13 - 5.18 | p = 0.912 | 0.3375 | -2.48 -3.15 | p = 0.011 |
| **Diameter at sinotubular junction** | 30.5 ± 4.6 | 31.8 ± 4.0 | 31.7 ± 3.7 | 1.27 | -2.84 - 5.37 | p < 0.001 | 0.1008 | -2.48 -2.68 | p = 0.404 |
| **Length of left leaflet** | 16.3 ± 2.5 | 16.3 ± 1.8 | 16.9 ± 2.1 | -0.23 | -4.31 - 3.86 | p = 0.820 | -0.6542 | -4.42 -3.12 | p < 0.001 |
| **Length of right leaflet** | 16.1 ± 2.1 | 16.1 ± 1.8 | 16.8 ± 2.2 | 0.04 | -3.47 - 3.54 | p = 0.972 | -0.7742 | -4.59 -3.04 | p < 0.001 |

CI = confidence interval; man = manual; mm = millimeter; SA = semi-automatic method; SA1 = semi-automatic method performed by readered 1; SA2 = semi-automatic method performed by readered 2; sign. = significance
